# Development of a conversation approach for practice nurses aimed at making shared decisions on goals and action plans with primary care patients

**DOI:** 10.1186/s12913-018-3734-1

**Published:** 2018-11-26

**Authors:** Stephanie Anna Lenzen, Ramon Daniëls, Marloes Amantia van Bokhoven, Trudy van der Weijden, Anna Beurskens

**Affiliations:** 1 Research Centre for Autonomy and Participation for People with a Chronic Illness, Zuyd University of Applied Sciences, Nieuw Eyckholt 300, 6419 DJ Heerlen, the Netherlands; 2 Department of Family Medicine, CAPHRI School for Public Health and Primary Care, Maastricht University, Maastricht, the Netherlands; 3 Assistive Technology in Healthcare, Zuyd University of Applied Sciences, Heerlen, the Netherlands

**Keywords:** Conversation approach, Shared decision making, Goal setting, Practice nurses, Primary care, Self-management

## Abstract

**Background:**

Primary care nurses play a crucial role in setting personal goals and action plans together with chronically ill patients. This may be a challenge for practice nurses, who are often trained to adopt protocol-based work routines. The aim of this study was to systematically develop a conversation approach, and a corresponding training course, for practice nurses aimed at making shared decisions about goals and actions with their chronically ill patients.

**Methods:**

The 6-step iterative Intervention Mapping protocol was used as a framework. This paper describes the first four steps of the protocol. After the first step, in which literature studies as well as qualitative studies were conducted, the overall aim and objectives for the approach were formulated (step 2). In step 3, methods and strategies for the approach were chosen, which were translated into practical components in step 4. In addition, a pilot study was conducted.

**Results:**

The main objectives of the approach focus on the ability of practice nurses to explore the patients’ perspectives from a holistic point of view, to explicitly formulate goals and action plans, to tailor shared decision making about goals and action plans to individual patients, and to continuously reflect on work-related attitudes. The approach consists of a practical framework for shared decision making about goals and actions. The framework involves a tool for exploring patients’ perspectives and a tool for identifying patient profiles, to facilitate tailoring shared decision making. A comprehensive training course for practice nurses was developed.

**Conclusion:**

We systematically developed a conversation approach, involving a practical framework with several tools, which aims to support practice nurses in making shared decisions about goals and actions with their patients. As practice nurses need support in their learning process to be able to share decisions with patients, we also developed a comprehensive training course for them. The approach and the training course were developed in close collaboration with important stakeholders. Some critical factors for the implementation of the approach were revealed. These factors will be addressed in the next step, a process evaluation (not part of this paper).

## Background

One of the major challenges for the primary care sector is the steady increase of people living with one or more chronic conditions, which has led to a growing interest in effective self-management support for patients within primary care [1, 2]. Self-management is defined as ‘the degree to which a chronically ill patient is able and willing to control his or her daily life’ [3, 4]. Self-management and self-management support involve not only medical management, but also maintaining and changing life roles (social self-management) and dealing with emotional consequences of the disease (emotional self-management) [5].

Self-management includes goal setting and action planning. Goal setting is a collaborative process of agreement on health-related goals between the health care professional and the patient [6]. Action planning is a collaborative process of agreeing on a course of actions to achieve a goal, addressing details such as what, when, where and how often’ [7]. Goal setting and action planning require professionals and patients to share decisions. The shared decision making model is widely recognized as a way to support patients in decision making [8]. It is defined as ‘an approach where clinicians and patients share the best available evidence when faced with the task of making decisions, and where patients are supported to consider options, to achieve informed preferences’ [8]. A widely used model for shared decision making is the three talk model developed by Elwyn et al. [8]. The three talk steps are: (1) choice talk: making sure to the patient that reasonable options are available, instead of just one option; (2) option talk: providing detailed information about options and (3) decision talk: considering preferences and deciding what is best. Shared decision making is thought to lead to more patient autonomy and may lead to better health outcomes [9]. Recently the shared decision making model is mostly applied in curative settings to make decisions about preference-sensitive treatment options. However, it may also be valuable in chronic care, as it highlights and explains the process of supporting patients in considering options and as it focuses on the collaboration between professionals and patients [10–13].

To put shared decision making into practice professionals need to have relational and communicative competencies (e.g., creating an environment for effective communication and interaction, listening to the patient, assessing the patient’s situation, including patient’s social environment and context) [14]. Moreover, professionals need to be able to self-reflect on their practice and stimulate patients to reflect on the process and outcomes of decision making throughout the whole process [15].

In routine care professionals often struggle to implement shared decision making (about goals and action plans). They experience it as time-consuming and complex and often struggle to elicit patients’ views and achieve mutual understanding and agreement about goals and action plans [16, 17]. Furthermore, professionals hesitate to involve patients in sharing decisions about goals and actions, as they assume that their patients are not able to make the right decisions [17–19]. There is currently a lack of practical frameworks and tools for supporting professionals in going through the process of shared decision making with patients in complex health care situations [10].

In Dutch primary care, as in many other countries, practice nurses play a role in supporting patients’ self-management. They see patients with chronic diseases on a regular basis and are mandated by the family physicians to apply most of the long-term condition management [20–22]. However, practice nurses in family medicine usually work according to fixed protocols that mainly focus on medical management. Their tasks often involve monitoring and recording disease-specific outcomes and they sometimes regard providing self-management support as something that increases their workload [23, 24]. This medical, protocol-based work routine conflicts with making shared decision about patients’ individual goals and action plans.

The primary aim of this study was to systematically develop a conversation approach, and a corresponding training course, for practice nurses working in primary care, aimed at making shared decisions about goals and actions with their chronically ill patients. The Intervention Mapping (IM) protocol was used as a guidance, as it provides a systematic process for development, implementation and evaluation [25].

## Development of the conversation approach

**Fig. 1:**
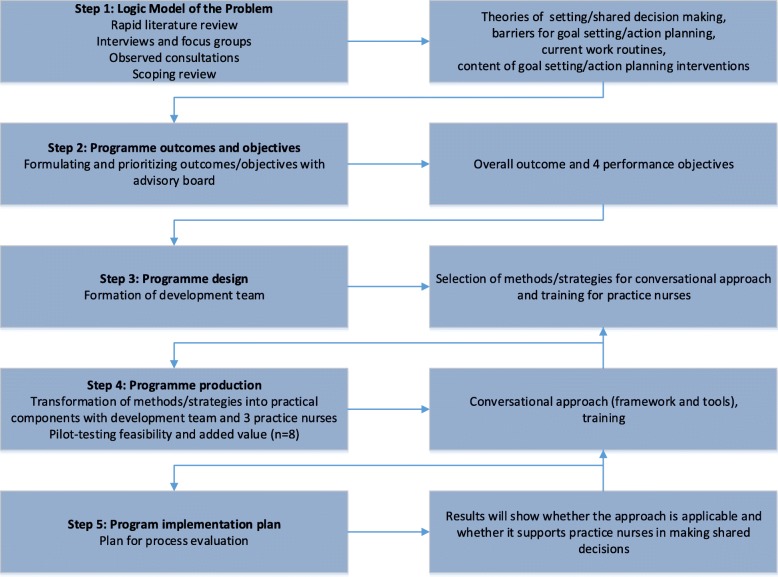
Overview of methods and results for steps one to five of the IM protocol

Intervention Mapping describes a six-step iterative process, with each step consisting of tasks that inform the next step. The six steps are: (1) logic model of the problem, (2) programme outcomes and objectives, (3) programme design, (4) programme production, (5) programme implementation plan, and (6) evaluation plan. The IM protocol highlights the involvement of key stakeholders in every step, as well as the importance of tailoring the approach to local needs and available resources. Moreover, theory and evidence are integrated throughout the whole process [25]. The present study involved steps one to four of the IM protocol and thereby focuses on the development process of the approach; the implementation plans and the evaluation of the approach are not part of this paper. The following section presents the methods and results of steps one to four. See Fig. 1 for an overview of the process, showing the methods and results for each step.

### Step 1: Logic model of the problem

The first step involved a needs assessment to identify what, if anything, needed to change and for whom. In order to gain an overview of theoretical assumptions, we first reviewed the literature about definitions, theories and models for shared decision making, goal setting/action planning, and self-management support [12]. We found that shared decision making models are mostly developed to support (medical) professionals to empower patients in making tailor-made treatment decisions [8]. These models often neglect the exploration and formulation of patients’ goals. However, in chronic care, setting goals for quality of life seems a precondition to prioritize relevant options for prevention, cure or care [10–13]. Theories and models for goal setting/action planning describe goal setting and action planning as an iterative process, consisting of several phases. The ‘Goal setting and Action Planning Practice Framework’, developed by Scobbie, Wyke & Dixon (2010) [26], is based on a comprehensive review of goal setting theories [27–29] and highlights the importance of exploring patients’ experiences before setting goals and then planning actions. The literature also frequently highlights that professionals need to act as coaches for their patients [20, 30–33]. A health coach seeks to support patients in setting goals that fit their situation and motivation, and health coaching is therefore tailored to what is important for patients [20, 30, 31].

Second, we explored experiences, needs, barriers and facilitators with regard to shared decision making about goals and action plans in primary care by conducting a descriptive qualitative study. Between April and June 2013, two focus groups and three individual interviews were conducted with patients, primary care professionals and experts (total participants = 17) [34]. The primary care professionals worked in practices for family medicine, occupational therapy, physical therapy, psychology and social work. The experts had experience of patient representation, self-management support, communication between patients and professionals, patient-centred practice and shared decision making. We found mostly barriers for the implementation of shared decision making about goals and action plans with chronically ill patients in primary care [34]. Barriers were related to the difficulties professionals encounter in exploring patients’ experiences from a holistic point of view (i.e. with regard to problems in everyday life, work, emotions, coping with the disease or support from the environment), and to explore patients’ goals and preferences and set medical or non-medical goals accordingly. Other barriers for goal setting/action planning in everyday practice were the professionals’ attitudes and skills with regard to involving patients in goal setting/action planning and the complexity of tailoring shared decision making about goals and action plans to the patients’ needs, motivation and capabilities. Moreover, time was considered a significant barrier [34].

Third, we examined the current working methods with regard to shared decision making about goals and action plans by observing eight consultations between practice nurses (*n* = 4) and patients (*n* = 8). After each observation we briefly interviewed (15 min) the practice nurse and patient about their experiences of the consultations. Practice nurses were all female and had at least one year of work experience; patients were aged between 75 and 89 years and all had complex (health) care requirements. Content analysis of the eight observed consultations revealed that explicit shared decision making about goals was rare. Action plans were frequently not made explicit and no agreement on actions was reached. Nurses often used a structured biomedical protocol (e.g. a structured questionnaire) to explore patients’ problems. They then initiated discussions about possible solutions to these problems. The interviews after the consultations showed that most patients were not aware of the purpose of the consultation and they could not recall any goals or actions agreed upon. Practice nurses found it difficult to reflect on their working method. Nevertheless, they were generally satisfied with the consultations. They reported a lack of guidance/tools to explore patients’ problems from a broader perspective.

Fourth, we conducted a scoping review in order to review the content of goal setting and action planning interventions in the context of self-management [35]. We identified 58 articles reporting on interventions for goal setting/action planning. By analysing the contents of the interventions we created an overview of phases, components and strategies for goal setting/action planning. We found that most interventions were disease-specific and focused on improving one or more predefined lifestyle behaviours. Although goal setting/action planning was recognized as a complex interactive activity, few of the interventions explicitly focused on communication or shared decision making about goals/action plans or on possibilities to tailor the intervention to patients’ needs, motivation and capabilities [35].

#### Conclusion step 1

To conclude, although theories and models about shared decision making, goal setting and action planning highlight the importance of (a) tailoring shared decision making about goals and actions to patients’ needs, motivation and capabilities, (b) viewing goal setting and action planning as one iterative process and (c) exploring patients’ experiences from a holistic point of view, professionals struggle to put this into practice. Moreover, professionals and patients experience difficulties in making goals explicit and experience a lack of time for goal setting. Nurses are not always aware of the process of shared decision making about goals and action plans and therefore find it difficult to critically reflect on their own working methods. Available interventions for goal setting/action planning mostly focus on changing lifestyle behaviour and less on a broader holistic perspective on goals or on communication and shared decision making about goals and actions.

### Step 2: Programme outcomes and objectives

Step two of the IM protocol identifies what should be targeted in the approach. Based on our needs assessment, the overall aim of the approach, as well as objectives, were formulated and prioritized by the advisory board of the project. The advisory board consisted of researchers, professionals or experts working for universities, primary care or patient organizations. The advisory board was closely involved in the project (from grant application to project implementation).

The overall goal of the approach was formulated as follows: practice nurses make shared decisions with their patients about goals and actions. Based on the needs assessment, we formulated four specific objectives: (1) practice nurses explore the patients’ perspectives from a holistic point of view (exploring medical symptoms, impact of the condition on everyday life/work, emotions, coping with the condition and support from the environment), (2) practice nurses explicitly formulate goals and action plans together with the patients (i.e. deciding together about goals and actions, as well as recording the goals and actions in the patient file), (3) practice nurses tailor shared decision making about goals and action plans to patients’ needs, motivation and capabilities (e.g. tailoring the communication about goals, the number and difficulty of goals and actions), and (4) practice nurses continuously reflect on their work routines and their work-related attitudes.

### Step 3 and 4: Programme design and producing of programme components

In the third step of the IM process, methods and strategies for the approach were chosen, which were translated into practical components for the approach in step 4 (producing programme components). Steps 3 and 4 were performed in an iterative process. This was done by a development team. The team consisted of patients, professionals and experts (on shared decision making, goal setting/action planning, self-management support, professional–patient communication, education and design) (*n* = 12). By involving experts from the Dutch National Health Care Institute and the Dutch College of General Practitioners we aimed to enhance national support for the project. During April 2014 and May 2015, the team met on a two-monthly basis. During step 4 (between January and June 2015) three practice nurses (two female, one male) were invited to closely collaborate with the development team. They were recruited from the researchers’ network and had over 10 years of work experience with chronically ill patients in primary care. They were asked to apply the approach, or parts of it, in their everyday practice and to share their experiences regarding the added value and feasibility with the development team. In monthly reflection meetings (*n* = 6), these practice nurses and the development team reflected on the experiences and further developed the approach. In addition, a pilot study was conducted to evaluate the added value and the feasibility of the approach. During January and April 2015, eight professionals (four female practice nurses collaborating with four male family physicians) were trained to use the approach and asked to apply it in practice over a period of eight weeks, with at least ten patients. After four weeks and after eight weeks, focus group interviews were conducted to explore participants’ experiences.

In steps 3 and 4 we first came up with a practical ‘framework for shared decision making about goals and action plans’, in which we combined an existing model for goal setting [26] with the three-talk model for shared decision making [8] (for further explanation see section titled ‘The conversational approach’). To support the use of the framework, we further developed a practical tool for exploring patients’ experiences, the ‘4-circles tool’ (for further explanation see section titled ‘The conversational approach’). For the pilot study we developed a training course, consisting of a 4-h workshop and a workbook. It aimed to support professionals in using the practical shared decision making framework and the 4-circles tool with their patients, and was presented by an experienced trainer, specialized in educating family medicine professionals. The training course included information on the background and theory of the approach, discussions of the approach, reflection about integrating the approach in everyday practice and role-playing exercises.

Through the close collaboration with the three practice nurses who had joined the development team and the pilot study we got more insights into the experiences with applying the framework and the 4-circles tool. We learned that all professionals experienced the approach as valuable in their everyday practice. The participants highlighted the value of being stimulated to explore patients’ experiences in greater depth. They felt that the practical shared decision making framework and the 4-circles tool led to more shared decisions and to more concrete goals/actions. However, most participants struggled to implement the approach in their everyday practice. They reported that they felt they lacked coaching skills. They needed more guidance to integrate the approach in their work routine and they experienced a lack of support for tailoring the approach to patients’ motivation and capabilities. They also hesitated to deviate from the commonly used biomedical protocol, as they felt it was their responsibility to ‘monitor’ the patients’ health status. They frequently found it difficult to deviate from their own professional ideas, as they worried that patients would make ‘wrong’ choices. They wanted more support in their learning process to become a coach for patients.

Based on these results we decided to (a) extend our conversation approach, in order to provide professionals with more support in tailoring the approach to individual patients and (b) develop a more comprehensive training course, to improve professionals’ coaching skills and change their attitudes. To this end, we expanded our development team by including a professional coaching company (Dubois & Van Rij), with extensive experience in developing training courses for professionals and which worked with an evidence-based patient profile model to support professionals in tailoring their communication to patients’ needs, motivation and capabilities.

## The conversation approach

The final conversation approach includes a practical ‘framework for shared decision making about goals and actions’.

### Practical framework for shared decision making about goals and actions

The practical framework for shared decision making about goals and actions forms the basis of the conversation approach. It aims to facilitate professionals in going through the process of shared decision making. It is based on and combines the three-talk model for shared decision making developed by Elwyn (1999) [8] and the ‘Goal setting and action planning practice framework’ developed by Scobbie, Wyke & Dixon (2012) [26]. The framework consists of 4 phases: Preparation, Goal setting, Action Planning and Evaluation (see Table 1). Each phase involves a number of steps that the professional can perform to achieve shared decision making with patients. Depending on the patient’s situation, the framework can be flexibly applied. The time spent on each phase can vary and patients and professionals can move back and forth between the phases in an iterative manner. In addition, supportive tools can be used in the different phases. A comprehensive workbook for the professionals explains the framework, its phases and steps, and offers examples of tools and strategies. Moreover, professionals are provided with printed descriptions of example cases and a video with an example case.

**Table 1:** Practical framework for shared decision making about goals and actions

Phase	Explanation
1 Preparation	Informing the patient about the aim of the consultation. Inviting the patient to ask questions or raise points for discussion.
2 Goal setting	
A Exploration	Exploring the patient’s current and desired situations.
B Giving Information	Giving information tailored to the patient.
C Formulating goals	Supporting the patient in formulating feasible goals.
3 Action planning	
A Choice talk	Making sure the patient knows that he/she has a choice.
B Option talk	Discussing possible options for actions with the patient.
C Decision talk	Deciding on actions together with the patient.
4 Evaluation	Continuously reflecting on the patient’s progress, and adjusting goals and actions.

This framework incorporates two tools: (1) a tool for exploring the patients’ perspectives (the 4-circles tool) and (2) a tool for identifying patient profiles in order to tailor shared decision making about goals and action plans to patients’ needs, motivation and capabilities. For the integration of the shared decision making framework and the two tools see Table 2.

**Table 2:** Integration of the practical framework for shared decision making with the tools

Practical framework for shared decision making about goals and action plans	Tools	
1 Preparation		
2 Goal setting A Exploration B Giving Information C Formulating goals	4-circles tool to explore the patient’s situation and goals	Patient profiles to adjust the communication and coaching to the individual patient
3 Action planning A Choice talk B Option talk C Decision talk		
4 Evaluation	4-circles tool to monitor and evaluate the patient’s goal achievement and to reset goals	

### Tool for exploring patients’ experiences: 4 –circles tool

**Fig. 2:**
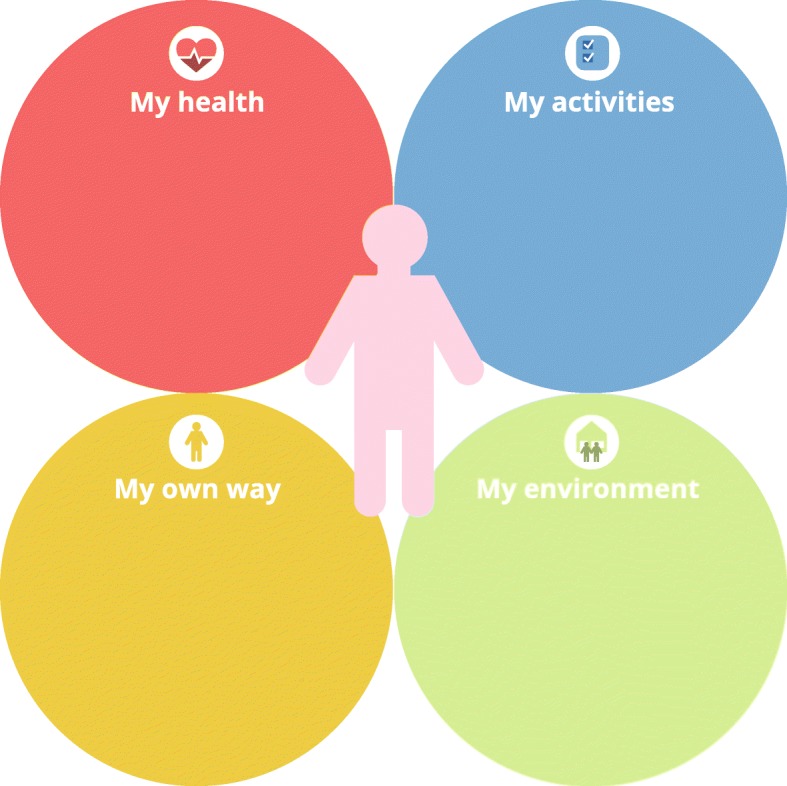
The 4-circles tool

The 4-circles tool (see Fig. 2) has been developed for use within the goal setting phase of the practical framework for shared decision making. It aims to support practice nurses and patients in collaboratively exploring the patients’ experiences with their condition from a holistic point of view and to facilitate a dialogue between nurse and patient about the patient’s current and desired situation (with regard to health, everyday life, social/physical environment and coping strategies). Its generic character means that the tool is not disease-specific and can be used for all chronically ill patients or other patients with complex health care requirements. It is meant to help patients gain insight into and reflect on their own situation, in order set goals (including non-medical goals) for them. The development of the 4-circles tool was inspired by A-FROM (Living with Aphasia: Framework for Outcome measurement), a tool to explore the impact of aphasia on a person’s everyday life [36]. Like A-FROM, the 4-circles tool is based on the framework of the International Classification of Functioning, Disability and Health (ICF), which presents a holistic approach to health, recognizing the interrelation between health and health-related domains [37]. The domains of the ICF have been simplified and visualized using four circles: (1) ‘My health’ represents the ICF domain of ‘Body Functions and Structures’. Within this circle, patients’ medical (physical and mental) symptoms are explored and patients are asked what they need to improve their symptoms. The questions and required professional actions from the practice nurses’ (biomedical) protocol can be asked and taken within this circle. (2) ‘My activities’ represents the ICF domains of ‘Activities’ and ‘Participation’. Patients are asked if they experience any difficulties in their everyday life activities or work activities, and if so, if they have already thought about solutions for these difficulties. (3) ‘My own way’ represents the ICF domain of ‘Personal Factors’. This circle includes questions about the way patients self-manage their condition, how they cope with the condition and what they need to improve their self-management. (4) ‘My environment’ represents the ICF domain of ‘Environmental Factors’ and explores patients’ physical and social environment, the support that patients get from their social environment and the degree of support that patients would like to get. Using this tool, the practice nurse can clarify the interrelationships among the circles (e.g. how does having problems in everyday life activities influence the patients’ self-management behaviour) and ask open-ended questions. Moreover, the tool can be used in a flexible way. The time spent discussing each circle depends on the patient, and the degree of support (e.g. the use of open or structured questions) can vary. Some patients can fill in the tool by themselves, others need more assistance. The tool can be printed on a A3 or A4 format. Notes can then be taken on the printed sheet and the patients can take it home.

### Tool for tailoring the use of the practical shared decision making framework: Patient profiles

**Fig. 3:**
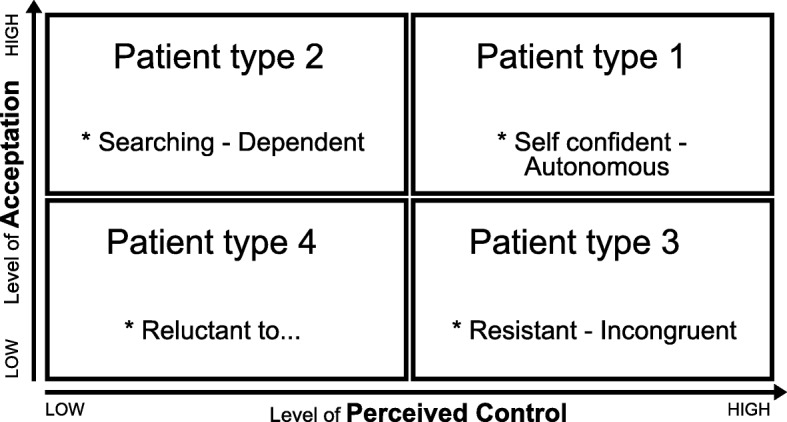
Patient profiles

To tailor the use of the practical framework for shared decision making about goals and action plans to the individual patients’ needs, motivation and capabilities we have developed a tool for identifying patient profiles (see Fig. 3). The tool is based on the theory-based patient typology by Bloem & Stalpers [38], which focuses on the role of the subjective experience of health as a motivator of patients’ health-related behaviours. The subjective experience of health is defined as ‘an individual’s experience of physical and mental functioning while living his life the way he wants to, within the actual constraints and limitations of individual existence’ [38]. As the typology focuses on patients’ experiences and motivation, it fits in well with the theoretical assumptions of shared decision making, goal setting and self-management support that are used for the conversation approach. Two key psychological determinants for the subjective experience of health have been identified by Bloem & Stalpers [38], and have been integrated in the patient profiles: (1) patients’ perceived control over the health condition and (2) patients’ acceptance of the health condition. Perceived control can be regarded as the patients’ belief that their health condition can be influenced or controlled by themselves or others. Acceptance can be interpreted as the patients’ feeling that their health condition and the possible consequences are acceptable for them personally. Based on a combination of these two determinants, four profiles have been identified, to support professionals in choosing actions and activities that may improve the patients’ subjective experience of their health [38]. As the determinants are dynamic constructs and a patient’s level of perceived control and acceptance can change with time and circumstances, patients do not have fixed positions [38].

For practical use, the four profiles have been translated into personas by Dubois & van Rij [39]. Typical behaviours for each persona have been described, as well as ways for practice nurses to adjust their communication (and the application of the practical shared decision making framework and the 4-circles tool) to the needs of each persona.

## The training course about shared decision making for practice nurses

The training course aims to improve practice nurses’ coaching skills with regard to making shared decisions with their patients and to stimulate them to continuously reflect on their work routines and their work-related attitudes. It consists of a one-day training session, individual on-the-job coaching (three weeks after the one-day training session) and a follow-up meeting (two months after the one-day training session). The trainer is a professional coach with 20 years of work experience. Throughout the training period (two months), the coach can be contacted by phone or email for questions or further advice. During the one-day training session, participants are introduced to the concepts of coaching and shared decision making and are trained to use the practical framework for shared decision making with the 4-circles tool and the tool for identifying patient profiles. The training is provided by means of information giving, discussions, role-plays and demonstrations of skills. In addition, participants are provided with a workbook containing information on the content of the training, as well as a link to a video demonstrating the use of the conversation approach. The individual coaching takes place three weeks after the one-day training session by means of a worksite visit. The coach observes two – four consultations of each practice nurse, and has a coaching session (30–60 min) with the nurse immediately after the consultations. The coach gives feedback on the nurse’s performance during or after the consultations, tailored to the nurse’s needs. The coaching can also involve role modelling. The coaching sessions also involve setting educational goals for the practice nurses. During the follow-up meeting with all participants (four hours), experiences are exchanged and role-plays and demonstrations of skills are provided. Overall, the training course uses training methods found to be effective for improving professionals’ skills. Role-playing and demonstrations of skills in actions, as well as constructive feedback from peers and skilled facilitators, have proved to be effective in improving practice nurses’ communication skills [40, 41]. The use of role modelling has proved to be important for professional development and an effective method for behaviour change [42]. Moreover, training at the nurses’ worksites is thought to enhance the integration of the skills into routine work [43]. By also focusing on exchanging experiences between practice nurses, the course intends to facilitate critical reflection on their work-related attitudes [44].

## Discussion

This paper has described the systematic development process and the content of a conversation approach and a corresponding training to help practice nurses working in primary care make shared decisions with their chronically ill patients about goals and actions. We came up with a practical framework for shared decision making about goals and actions, and two tools to support the use of the framework (a tool for exploring the patients’ perspective and a tool for identifying patient profiles in order to tailor goal setting/action planning). We also developed a training course for practice nurses, focusing on the coaching skills and attitudes needed to put the approach into practice.

A strength of our conversation approach is the combination of the traditional medically focused shared decision making model with the goal setting framework [8, 26]. Most of the existing models for shared decision making have been developed for the purpose of making medical treatment decisions and are difficult to apply in chronic care [10]. Within chronic care, the desired health state and the patients’ goals are frequently less clear and will differ between individuals and at different points in time [45]. Patients can experience and define their health differently than from the medical viewpoint, and frequently also have non-medical goals [46, 47]. For most chronically ill patients, dealing with emotional reactions (emotional self-management) and adjusting social roles (social self-management) are as important as dealing with medical instructions and lifestyle recommendations (medical self-management) [34, 48, 49]. Therefore, patients’ goals need to be explored explicitly [46, 47]. A goal setting phase prior to the phase of making shared decisions about actions is therefore indispensable in chronic care.

Another strength of the approach is its flexible character, as the process may vary regarding the amount of time spent on the different phases of shared decision making, the degree of support professionals provide to patients and the tools that professionals can use within each phase. As the experiences of health and quality of life of chronically ill patients and their goals vary over time, the framework highlights the importance of regularly exploring the patients’ experiences and constantly adapting to each individual patient [46, 47]. In order to support professionals in doing this, our conversation approach includes two easy-to-use tools. Practical tools for shared decision making are thought to facilitate overcoming the difficulties professionals experience in sharing decisions with patients [18].

However, during the development process we revealed some critical factors for the implementation plan of the approach. It became clear that the professionals struggled to integrate the framework and the tools into their everyday practice. They needed more guidance to integrate the framework into their existing fixed protocols and work routines. Although they appreciated the tools, they hesitated to deviate from their current working methods and their own professional ideas about ‘what’s best for the patient’. The practice nurses seemed to struggle with a professional role conflict. Faced with the changing role for nurses in primary care (from medical expert to coach), nurses may feel uncertain about their professional identity [50]. Nurses’ professional identity is defined as ‘the values and beliefs held by nurses that guide their thinking, actions and interactions with the patient’ [44, 51]. A professional identity is developed and shared among members of a profession, through training, qualifications and socialization [48]. It reflects the nurses’ professional self-concept, and hence their own beliefs about their roles, values and behaviours, as well as the public’s image of the profession [44].

In the needs assessment phase of our study, especially in the qualitative studies, we concentrated on experiences and problems with regard to shared decision making and goal setting for practice nurses in primary care. We identified the influence of the skills and attitudes the professionals need for shared decision making, and obtained less information about the difficulties practice nurses experience in changing their role in primary care, i.e. adjusting their professional identity. These difficulties emerged during the pilot studies, and we therefore developed a more comprehensive training course for practice nurses, also incorporating individual on-the-job coaching, in which practice nurses can reflect on their professional self-concept with an experienced coach. Additionally, we incorporated more opportunities for exchange between practice nurses and for reflections about professional identity, as interaction with other nurses and sharing experiences in a reflective way was found to contribute to the development/adjustment of professional identity [44].

Nonetheless, when aiming to implement the approach it is essential to pay more attention to integrating the approach into the structured protocols and to the nurses’ attitudes towards shared decision making. A necessary future step is to make an implementation plan that focuses on processes and conditions that facilitate the integrating of shared decision making in routine care and tailoring training to the individual professional’s learning needs.

As regards the development process, we think that it is a strength that we involved different stakeholders, as well as experts in different fields, professionals and patients throughout the process. We developed, evaluated and adapted the approach together with future users (practice nurses), and used the IM protocol as a guideline. While the steps of the IM protocol are described in a linear order, the planning process is, in fact, iterative [25]. However, during the process of development we pilot-tested and adjusted the approach only in step four of the IM protocol. More frequent testing of the approach in several shorter iterative cycles, for example by combining the IM protocol with user-centred design methods, would probably have given us more information about the feasibility of the approach in practice.

## Conclusions

We systematically developed a conversation approach, consisting of a practical framework with several tools that aim to support practice nurses in making shared decisions on goals and actions with their patients. As practice nurses need support in their learning process to be able to share decisions with patients, we also developed a comprehensive training course for nurses. We developed the approach and the training course in close collaboration with major stakeholders. During the development process some critical factors for implementation of the approach were revealed. These critical factors will be addressed in the process evaluation plan, aiming to evaluate the feasibility and added value of the approach and the training course in routine primary care.
